# A protein network-guided screen for cell cycle regulators in Drosophila

**DOI:** 10.1186/1752-0509-5-65

**Published:** 2011-05-06

**Authors:** Stephen T Guest, Jingkai Yu, Dongmei Liu, Julie A Hines, Maria A Kashat, Russell L Finley

**Affiliations:** 1 Center for Molecular Medicine and Genetics, Wayne State University School of Medicine, Detroit, Michigan, 48201, USA; 2 National Key Laboratory of Biochemical Engineering, Institute of Process Engineering, Chinese Academy of Sciences, Beijing, 100190, China; 3 Department of Biochemistry and Molecular Biology, Wayne State University School of Medicine, Detroit, Michigan, USA, 48201 and Karmanos Cancer Institute, Detroit, Michigan, 48201, USA

## Abstract

**Background:**

Large-scale RNAi-based screens are playing a critical role in defining sets of genes that regulate specific cellular processes. Numerous screens have been completed and in some cases more than one screen has examined the same cellular process, enabling a direct comparison of the genes identified in separate screens. Surprisingly, the overlap observed between the results of similar screens is low, suggesting that RNAi screens have relatively high levels of false positives, false negatives, or both.

**Results:**

We re-examined genes that were identified in two previous RNAi-based cell cycle screens to identify potential false positives and false negatives. We were able to confirm many of the originally observed phenotypes and to reveal many likely false positives. To identify potential false negatives from the previous screens, we used protein interaction networks to select genes for re-screening. We demonstrate cell cycle phenotypes for a significant number of these genes and show that the protein interaction network is an efficient predictor of new cell cycle regulators. Combining our results with the results of the previous screens identified a group of validated, high-confidence cell cycle/cell survival regulators. Examination of the subset of genes from this group that regulate the G1/S cell cycle transition revealed the presence of multiple members of three structurally related protein complexes: the eukaryotic translation initiation factor 3 (eIF3) complex, the COP9 signalosome, and the proteasome lid. Using a combinatorial RNAi approach, we show that while all three of these complexes are required for Cdk2/Cyclin E activity, the eIF3 complex is specifically required for some other step that limits the G1/S cell cycle transition.

**Conclusions:**

Our results show that false positives and false negatives each play a significant role in the lack of overlap that is observed between similar large-scale RNAi-based screens. Our results also show that protein network data can be used to minimize false negatives and false positives and to more efficiently identify comprehensive sets of regulators for a process. Finally, our data provides a high confidence set of genes that are likely to play key roles in regulating the cell cycle or cell survival.

## Background

The discovery of RNA interference (RNAi) has revolutionized the way in which loss-of-function studies can be performed [1]. Activation of RNAi using double-stranded RNA (dsRNA) that targets a transcript induces destruction of the transcript and a corresponding reduction in the expression level of the encoded protein(s). Genome-wide RNAi libraries that allow for efficient knockdown of virtually any gene are now available for studying organisms ranging from *C. elegans *to human [2-7]. These libraries have opened the door for large-scale RNAi-based screens aimed at identifying genes involved in a wide variety of cellular processes. Completed screens have successfully identified novel regulators of cell growth and viability [4,7-10], signaling pathways [11-16], cell morphology and the cytoskeleton [17-21], pathogen infection [15,22-24] and many other important cellular processes [25-27]. In some cases, the same cellular process has been examined by more than one independent screen. Surprisingly, comparing the results of similar screens has revealed a low level of overlap in the genes that are identified [28-33]. This low level of overlap suggests that these large-scale RNAi screens result in high numbers of false positives and false negatives, though the relative rates at which these are produced are largely unknown. The unknown rate of false positives raises questions about how to best interpret the data and what level of validation is required. The rate of false negatives on the other hand, limits the extent of information that can be derived from a large-scale screen for any biological process.

One potential source of false positives in RNAi-based screens comes from off-target effects that occur when a dsRNA contains homology to mRNAs other than the target mRNA. This can result in reduced expression of non-target genes and an incorrect association between the intended target gene and a phenotype. In cultured cells of the model organism Drosophila where long (300-800 bp) dsRNAs are routinely used for inducing RNAi, off-target effects have been shown to be prevalent [34,35]. Off-target effects have also been shown to be present in human cells where smaller siRNAs are used [36-38]. Improvements in the design of RNAi reagents have helped minimize off-target effects but they have not eliminated the problem [25,39]. One experimental approach to identifying potential off-target effects involves testing multiple dsRNAs for each gene identified as a hit in a large-scale screen. Multiple dsRNAs that are homologous to different regions of a gene but not to each other are unlikely to affect the same non-target genes. Therefore, if two or more unrelated dsRNAs targeting the same transcript display the same phenotype it is likely that this phenotype is the result of knockdown of the intended target gene and not the result of an off-target effect. When large-scale, RNAi-based screens first became possible the importance of controlling for off-target effects had not been fully appreciated. As a result, many of the early screens did not test multiple dsRNAs for each hit and are, therefore, likely to contain significant numbers of false positives. In order to confirm the results of these early studies, the hits that were identified in these screens need to be re-examined using additional dsRNAs.

One source of false negatives in large-scale RNAi-based screens is the inefficient knockdown of specific target genes by some of the RNAi reagents under the conditions used. The efficiency of knock down can be directly monitored by quantitative assessment of target gene mRNA or protein levels, but this is rarely practical for a genome-wide or other large-scale screen. Another way to gain increased confidence for a negative result is to confirm it in an independent study, for example, using a different RNAi reagent for the gene or different screening conditions. However, because the number of genes that are scored as negatives in a large-scale screen typically far exceeds the number of genes that give a positive result, it is more difficult to independently confirm the set of negatives. It is also likely that the majority of the negative genes are indeed true negatives and therefore re-screening the entire set of negatives would be an inefficient approach. A more efficient approach for identifying false negative results may be to re-screen a subset of genes that were first enriched for potential positives, similar to the approach proposed for generating comprehensive interactome maps [40]. The information that is now available in gene and protein interaction maps has the potential to serve as a guide for identifying such subsets of genes.

Virtually all cellular processes rely on specific physical interactions between proteins. As a result, groups of proteins that regulate a particular cellular process tend to be closely connected to each other in the protein interaction network [41-51]. By searching protein interaction data for the partners of known regulators of a process, it is possible to identify new regulators of the process [41,52-54]. This simple 'guilt-by-association' approach has been used to successfully predict the functions of novel proteins, as have more sophisticated analyses of protein interaction networks [55-58] (reviewed in [59]). Since these approaches use interaction data to predict which proteins are involved in a process, they could also predict which negatives from a large-scale RNAi screen are most likely to be the false negatives. Independently confirming or re-screening this group of genes is a potentially efficient approach for identifying all of the genes that are involved in a particular cellular process.

One important cellular process that has been examined by multiple large-scale RNAi-based screens is the cell division cycle [30,31,60,61]. In Drosophila, two screens aimed at identifying cell cycle regulators have been performed by treating cultured S2 cells with dsRNAs targeting a large number of different genes and screening for cell cycle defects by flow cytometry. One screen used dsRNAs targeting each of the Drosophila protein kinases (i.e., the kinome) [61] while the other screen used dsRNA targeting most genes [60]. The kinome and genome-wide screens identified 41 and >400 putative cell cycle regulators, respectively. Among the commonly screened genes, only 24 were identified in both, while 17 were uniquely identified in the kinome screen and 19 were uniquely identified in the genome-wide screen. Neither study confirmed hits with multiple dsRNAs to guard against off-target effects. In the current study, we performed an independent screen examining cell cycle regulators that were identified in one or both of the two previous large-scale screens. We also examined a subset of the negatives from those screens that we identified as possible false negatives based on protein interaction data. Our results confirm many of the originally identified cell cycle phenotypes, identify previously unknown cell cycle regulators, and establish protein interaction map data as an effective tool for guiding RNAi-based screens and for reducing false positive and negative results.

## Results

### A virtual protein-protein interaction screen

**Figure 1:**
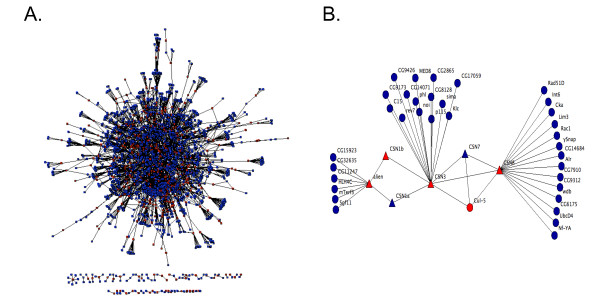
**A virtual protein-protein interaction screen**. (A) 642 Drosophila putative cell cycle regulators were used as baits to query DroID, the Drosophila Interactions Database. Baits included hits from the two previous RNAi-based screens for cell cycle regulators along with a set of additional genes annotated as being involved in the cell cycle. The original interaction map was filtered to remove low confidence interactions (see Methods). The filtered map includes 473 of the bait proteins (red nodes), and 1843 interactors (blue nodes). 94.8% of the proteins are connected into one large network. (B) A subnetwork from (A) involving six members of the COP9 signalosome protein complex that was previously identified as a regulator of the G1/S transition. Signalosome components are shown as triangles while their interaction partners are circles. Proteins that were used as baits in the virtual protein-protein interaction screen are shown in red while their interaction partners are shown in blue.

The results of two large-scale, RNAi-based screens in cultured Drosophila cells have identified genes that are potential regulators of the cell cycle [60,61]. We set out to provide independent confirmation of the identified regulators, and to identify potential false negatives from the previous screens. To identify a subset of the negative genes that were likely to be enriched for cell cycle regulators, we performed a virtual protein-protein interaction screen to find proteins that interact with known or suspected cell cycle regulators. The bait proteins that we used for the virtual screen were proteins identified as potential cell cycle regulators in the two published RNAi-based screens as well as all genes annotated with a Gene Ontology (GO) biological process [62] of "cell cycle" (see Methods). These 642 bait proteins were used to query DroID, the Drosophila Interactions Database [63,64] to identify 5,008 potential protein interaction partners (Additional File 1). We filtered this data (see Methods) to obtain a higher confidence set that consisted of 1,843 interaction partners for the 642 bait proteins (Figure 1A and Additional File 1). We hypothesized that the interaction partners of the baits would be enriched for cell cycle regulators relative to random proteins. In support of this, analysis of both the filtered and unfiltered protein network showed that the bait proteins interact with each other much more than would be expected for a random group of proteins (p-value < 10^-82^) (Additional File 2A). The high level of connectivity between bait proteins is also evident from the size of the maximally connected component subnetwork for the baits, which was found to be significantly larger than for equally sized random sets of proteins (p-value < 10^-18^) (Additional File 2B). This analysis demonstrates that within the protein interaction data that we screened, cell cycle regulators frequently interact with each other. It also supports the hypothesis that proteins that interact with the bait proteins that we used in the virtual screen may be enriched for novel cell cycle regulators that were false negatives in the previous screens. Figure 1B shows a subset of the interaction map data involving 6 members of the COP9 signalosome protein complex that was identified as a regulator of the G1/S transition in one of the previous screens [60]. As expected for a protein complex, there are a number of interactions that connect COP9 signalosome subunits to each other in the map. There are also a number of interactions between COP9 signalosome subunits and non-complex members. These interactors potentially function in conjunction with the COP9 signalosome to regulate cell cycle progression and represent possible false negatives from previous screens.

### A directed RNAi screen for cell cycle regulators in Drosophila cells

**Figure 2:**
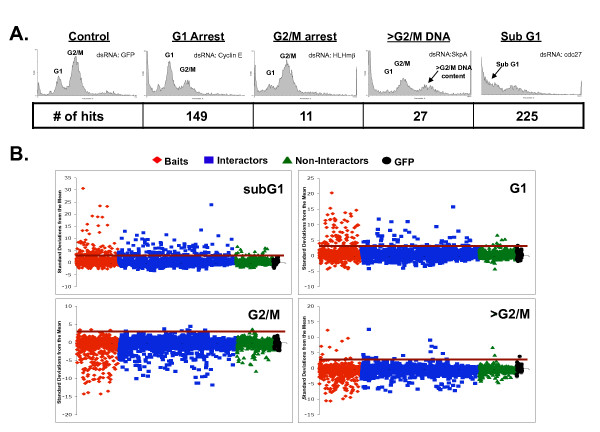
**Cell cycle defects induced by dsRNA**. (A) Examples of cell cycle profile phenotypes. Each panel shows the cell cycle profile for Drosophila S2R+ cells that have been treated with the indicated dsRNA. The location of cell populations with G1 and G2/M DNA content is labeled. Cell populations with >G2/M or subG1 DNA content are indicated in panels where there is a significant increase in these cell populations. The number of dsRNAs that displayed an increase of greater than 3 standard deviations from the mean is shown below each phenotype (# of hits). (B) dsRNA targeting cell cycle baits and their interactors cause strong cell cycle defects more frequently than dsRNA targeting other proteins. The percentage of cells in each cell cycle phase following treatment with individual dsRNAs was determined and the number of standard deviations that this value differs from the mean was plotted. Dot plots show data for dsRNAs targeting baits (red diamonds), interactors of the baits (blue squares), non-interactors (green triangles), and GFP (black circles). The red horizontal line in each panel is drawn at 3 standard deviations from the normalized mean of control dsRNA. The genes above the line were considered as hits, or potential cell cycle regulators.

**Figure 3:**
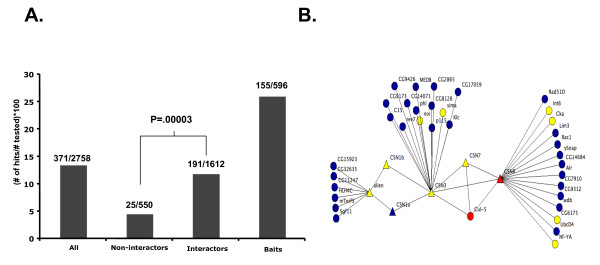
**The interaction map-guided approach improves hit rate in an RNAi screen and identifies novel cell cycle regulators**. (A) Hit rate for the different classes of genes in the screen. Hit rate was determined as the percentage of genes that scored as a hit from the total number of genes tested for that class. (B) COP9 signalosome interaction map from Figure 1B showing genes identified as hits in the current study (yellow). These included a number of genes not previously identified as hits in RNAi screens for cell cycle regulators (yellow circles).

We used a previously described library of dsDNA templates [3,65] that allowed for the generation of dsRNAs targeting 596 of the 642 bait protein genes and 1,612 of the 1,843 interaction partner genes. We also generated dsRNAs targeting a random set of 550 genes encoding proteins that were not known to interact with the cell cycle baits or their interactors (Methods). We treated Drosophila S2R+ cells with dsRNAs targeting a total of 2,758 genes and determined cell cycle profiles by flow cytometry. Cell cycle profiles were used to determine the percentage of cells with G1, G2/M, greater than G2/M, or less than G1 DNA content (Additional File 3). dsRNAs that induced a significant increase (>3 standard deviations from the mean) in the percentage of cells in any of these four categories were considered as hits. Examples of cell cycle phenotypes are shown in Figure 2A. Overall, the screen identified 371 unique genes as hits (Additional File 4). A global view of the data reveals that the majority of the strong phenotypes were observed for dsRNAs targeting the putative cell cycle proteins that we used as baits or their interaction partners (Figure 2B). As expected, targeting of the baits resulted in the highest rate (26.0%) of cell cycle defects (Figure 3A). The hit rate for bait interaction partners was 11.8%, significantly higher than the hit rate for the set of random non-interactors (4.5%) (Figure 3A). This was also true for interactors that were derived just from baits that were hits in previous screens without regard to their Gene Ontology annotation. For those, 12.3% (160/1303) were hits, suggesting that prior knowledge of the Gene Ontology annotation of the baits was not necessary to enrich for hits over random genes. Additionally, genes from the group of interactors that were hits interacted with a greater number of baits than did the interactors that were non-hits (p = < .0001) (Additional File 5). We also found that the quality of the protein interaction data affected the hit rate in our RNAi screens. For example, interactors connected to baits by higher confidence interactions [66] were more likely to be hits than those connected by low confidence interactions (Additional File 6). Interestingly, the hit rate for non-interactors was similar to the hit rate observed in undirected genome-wide screens [4,60]. These results show that protein interaction map data can be used to identify a set of genes that is enriched for regulators of a cellular process like the cell cycle. Moreover, the identification of phenotypes for a substantial number of genes that were negative in previous screens shows that the interaction map-guided approach can help to identify putative false negatives from the hits reported in individual screens (e.g., see Figure 3B).

### Validation of Identified Cell Cycle Regulators

**Figure 4:**
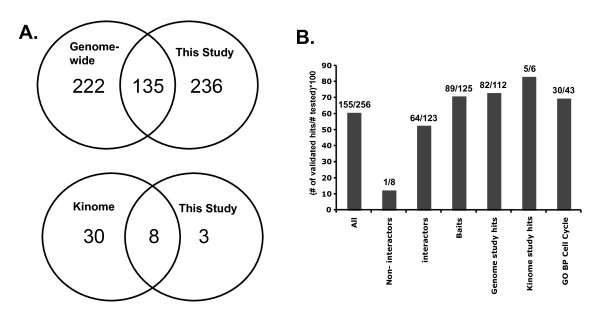
**Comparison of genes identified in this study and two previous large-scale RNAi-based screens for cell cycle regulators**. (A) Venn diagrams showing the overlap between hits identified in the current study and those identified by Bjorklund et al. in a genome-wide screen [60] and by Bettencourt-Dias et al. in a kinome-wide screen [61]. Only genes screened in common between each pair of screens being compared are included for this analysis. Only genes that displayed a flow cytometry phenotype in Bettencourt-Dias et al. were used. (B) Validation rates for genes that we analyzed with a second dsRNA. Also shown is the validation rate for genes annotated with Gene Ontology biological process (GO BP) "cell cycle" [62]. Validation rate was determined as the percentage of genes that were tested with a second dsRNA that showed a matching phenotype for both dsRNAs.

Comparison between the results of previous screens and the current screen revealed a significant overlap in the genes that were identified as hits (Figure 4A). Of the 375 hits from previous screens that were re-screened in the current study, 138 or 36.8% were identified as hits in the current study. However, our screen and the previous cell cycle RNAi screens each identified substantial numbers of unique hits. We consider three possible explanations for this lack of overlap. First, it is possible that the screens have different levels of sensitivity and that one screen captured a larger fraction of the true positives than the other. This seems unlikely given that each screen (e.g., ours and the genome-wide screen) had similar numbers of hits (371 vs. 361) among the genes screened in common (Figure 4A). Second, the lack of overlap may be due to high but roughly equal rates of experimental false negatives in each screen. Such false negatives, for example, could result from inefficient knock down of gene expression by specific dsRNAs. A third possibility is that many of the novel hits in each screen are the result of off-target effects. This possibility must be given serious consideration since the previous screens did not control for off-target effects and, in at least the genome-wide screen, full-length cDNAs were used to produce dsRNA [60]. dsRNA generated from full-length cDNAs are more likely to lead to off-target effects than dsRNA from smaller regions of each transcript [35]. We performed GO enrichment analysis [67] on the hits from each dataset (Additional File 7). As expected, hits from each dataset were significantly enriched for cell cycle regulators. Moreover, the hits in common between our screen and the previous genome-wide screen were significantly more enriched for cell cycle regulators than the unique hits, consistent with the idea that the overlapping data is enriched for true positives. However, the hits that were unique to each screen were also significantly enriched for cell cycle regulators (p values < 10^-4 ^and <10^-16^, respectively), indicating that each screen detected true positives that were false negatives in the other screen (Additional File 7). Interestingly, our set of novel hits was more enriched for cell cycle regulators than the novel hits from the previous genome-wide screen, possibly because we used smaller dsRNAs that are less prone to off-target effects.

To confirm the putative novel cell cycle regulators that we identified and to determine the rate at which they may be due to off-target effects, we generated validation dsRNAs for 256 genes. The validation dsRNAs targeted regions of the transcripts not overlapping or only minimally overlapping (<12 nucleotides) with those targeted by the dsRNA used in the initial screen. Overall, 155 (60.5%) of the hits from the primary screen were validated by testing an additional dsRNA (Figure 4B; Additional File 3; Additional File 4). This validation rate is similar to that of other validated RNAi-based screens for regulators of other biological processes in cultured Drosophila cells [68-70]. The validation rate for hits identified in both the current screen and a previous screen was significantly higher than for genes identified only in the current screen (72.8% versus 50.7%) further suggesting that genes identified in two, independent screens are more likely to be true positives than genes identified in only a single screen. This conclusion is also supported by a repeat screen in which we re-screened 4 plates of dsRNAs from the original screen a second time (Additional File 3). Of the 52 hits identified in the initial screen, 36 were identified again in the repeat screen. Hits identified in both the initial screen and the repeat screen were more likely to be confirmed by a validation dsRNA (24/33 versus 2/7). Combined, these results suggest that one major reason for the low level of overlap among different RNAi screens that probe the same biological process is the prevalence of off-target effects, which can be as high as 40-50% in any given screen. This is consistent with other recent studies and further highlights the importance of validating genes identified in RNAi screens, as previously recommended [34,35,71].

Our results also suggest that false negatives are prevalent in individual RNAi screens. Among the validation dsRNAs that we tested, 142 targeted genes were hits in our initial screen but not in previous screens. 72 (50.7%) of these validation dsRNAs confirmed the results of the primary screen (Additional File 3). This finding indicates that these genes were false negatives in the previous screens. Combining the complete set of validated genes from the current study with genes that were identified both in the current screen and a previous screen (regardless of validation in current screen) allows us to define a set of 210 genes that are high confidence regulators of the cell cycle (Additional File 4).

### New cell cycle regulators

Among the 155 validated hits from the current study, 46 were neither classified as cell cycle genes (based on Gene Ontology identifier GO:0007049 [62]) nor were they hits in one of the previous RNAi-based screens [60,61]. These genes are therefore novel cell cycle regulators that were identified in the current study. Thirty-three of the identified genes displayed a subG1 phenotype, 11 displayed a G1 phenotype, 1 (*zipper*) displayed a greater than G2/M phenotype, and 1 displayed both a G1 and sub-G1 phenotype (Additional File 4). The most common ontology associated with the 33 genes that displayed a sub-G1 phenotype was mRNA processing (11/33). This finding suggests that mRNA processing plays a critical role in maintaining viability in these cells. A similar dependence on mRNA processing for cell viability has been observed in other RNAi-based screens in Drosophila cells as well as in studies of *S. cerevisiae *[72,73], *C. elegans *[2,74] and human cells [75]. The single gene with a greater than G2/M DNA content phenotype was the Drosophila non-muscle Myosin II gene *zipper*. *Zipper *has been identified in two previous large-scale RNAi-based screens for cytokinesis regulators [65,76] and has an established role in the process of cytokinesis (reviewed in [77]). Among the 11 novel G1 regulators there were two genes, *Rae1 *and *l(2)dtl*, encoding proteins with WD40 repeat domains [78,79]. Neither gene had been annotated with any GO terms for molecular function or biological process. A literature search, however, revealed a previous study that focused on *Rae1 *and cell cycle regulation [79]. Interestingly, this study also identified a G1 phenotype for *Rae1 *following RNAi in cultured Drosophila cells [79]. In the case of *l(2)dtl*, while the Drosophila gene had not been annotated as a cell cycle regulator, data from both Drosophila and human cells suggest that the l(2)dtl protein (L2DTL) is a targeting subunit of the CUL4/DDB1 ubiquitin ligase complex that targets critical cell cycle regulators for degradation [80]. RNAi targeting the human ortholog has been shown to cause growth arrest and an increase in both p53 protein and the DNA replication licensing factor CDT1 [81,82]. Additionally, human *l(2)dtl *has been shown to oscillate during the cell cycle with peak expression occurring at the G1/S transition, consistent with a role in regulating G1/S [83]. The remaining genes that displayed a G1 phenotype contained multiple members of three structurally related protein complexes (see below).

### Three related protein complexes regulate the G1/S cell cycle transition

**Figure 5:**
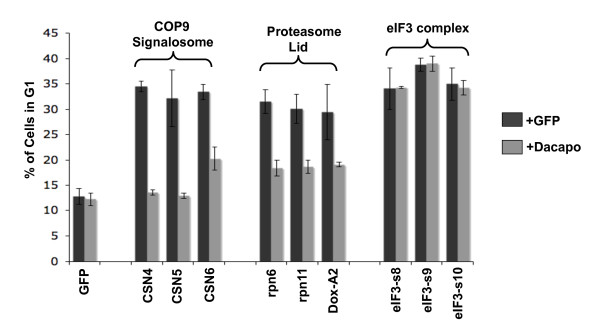
**Suppression of G1 arrest by simultaneous knockdown of Dacapo is protein complex-specific**. S2R+ cells were treated with dsRNAs targeting the indicated members of the COP9 signalosome, proteasome lid or eIF3 protein complex in combination with either dsRNA targeting GFP (light grey) or Dacapo (dark grey). The percentage of cells with G1 DNA content was determined by flow cytometry. Error bars are standard error of three replicates. Results for additional subunits of these complexes as well as other genes that produced a G1 arrest upon knock down are shown in Additional File 8.

An examination of genes in the list of 210 high confidence cell cycle regulators that displayed a G1 phenotype revealed the presence of multiple members of three structurally related protein complexes: the eukaryotic translation initiation factor 3 complex, the COP9 signalosome, and the proteasome lid. These three complexes have been referred to as "the zomes" based on their related structures. Each complex is composed of protein subunits that contain a domain named PCI (proteasome-COP9-eukaryotic initiation factor) and protein subunits that contain a domain named MPN (Mpr1-Pad1 N-terminus) [84,85]. We sought to identify mechanisms that underlie the G1 phenotype observed following RNAi targeting each of these complexes. Progression of cells through the G1 phase of the cell cycle requires activation of the Cyclin dependent kinase 2 (Cdk2) and Cyclin E (CycE) complex [86-92]. Drosophila Dacapo (Dap), a member of the p21^CIP1^/p27^KIP1 ^family of Cdk inhibitors, can block progression from G1 into S phase by specifically inhibiting Cdk2/CycE [93,94]. To determine if Dap mediates the G1 arrest induced by RNAi targeting the zomes, cells were treated with dsRNA targeting members of each zome complex alone or in combination with dsRNA targeting the Dap transcript (Figure 5 and Additional File 8). We observed that Dap knockdown completely rescued the G1 arrest induced by RNAi targeting members of the COP9 signalosome, consistent with a similar demonstration by Bjorklund et al. [60] for CSN1b, CSN2 and CSN5. Knockdown of Dap also completely rescued the G1 arrest caused by targeting subunits of the proteasome lid. The COP9 signalosome and proteasome lid complexes both play a role in ubiquitin-mediated proteolysis of Dap/p21/p27 in human and Drosophila cells [95-97]. RNAi targeting the proteasome lid or COP9 signalosome, therefore, likely stabilizes Dap leading to increased Cdk2 inhibition and delayed progression from G1 into S phase. Interestingly, dsRNA targeting Dap transcripts did not have a significant effect on the G1 arrest induced by knocking down members of the eIF3 complex (Figure 5 and Additional File 8). These results suggest that the G1 arrest induced by knocking down eIF3 subunits is not mediated by Dap, or not solely by Dap, and that the underlying mechanism is distinct from that which mediates the COP9 signalosome and proteasome lid phenotypes.

There are several lines of evidence that have previously implicated the eIF3 complex in regulation of the G1/S transition in other organisms. In the yeast *S. cerevisiae*, a cell division cycle mutant, *cdc63*, that arrests in G1 encodes a component of the eIF3 complex [98-101] and is the ortholog of Drosophila eIF3-S9 that was identified in this study. Temperature sensitive mutants of TIF34, another yeast eIF3 component, also arrest in G1 at the restrictive temperature [102,103]. The Drosophila TIF34 ortholog was not screened in this study but was a negative in the previous genome-wide screen [60]. In human tissue culture cells, overexpression of 5 different eIF3 proteins each resulted in an increase in the percentage of cells in S phase and an increased rate of cell proliferation suggesting that the human eIF3 complex also regulates G1/S and cell cycle progression [104]. Together with our results, these studies indicate an evolutionarily conserved role for the eIF3 protein complex in regulating the G1/S transition. The mechanism by which the eIF3 complex regulates the G1/S transition of the cell cycle however is not known, but appears to be independent of Dap regulation.

### RNAi directed against members of the eIF3 protein complex does not affect Cyclin E expression but does affect Cyclin E associated kinase activity

**Figure 6:**
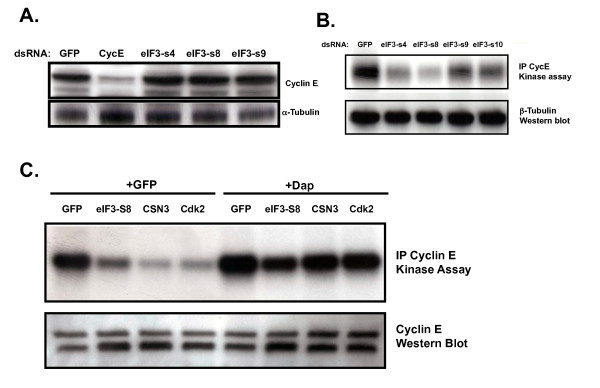
**RNAi directed against members of the eIF3 protein complex does not affect Cyclin E expression but does affect Cyclin E-associated kinase activity**. (A) Western blot for Cyclin E expression in whole cell extracts from S2R+ cells treated with the indicated dsRNAs. (B) Kinase activity on histone H1 of Cyclin E immunoprecipitates from S2R+ cells treated with the indicated dsRNAs. (C) Kinase activity on histone H1 of Cyclin E immunoprecipitates from S2R+ cells treated with dsRNA targeting the indicated members of the COP9 signalosome, proteasome lid or eIF3 protein complex in combination with either dsRNA targeting GFP or Dacapo.

Activation of *CycE *transcription, increased CycE protein expression, and the activation of Cdk2 by CycE are all required for cells to progress from G1 into S phase [87,105]. eIF3 is a large, multi-subunit complex that has been shown to play a key role in regulating mRNA translation and thus gene expression [106]. One possible mechanism by which eIF3 could be required for G1/S is that eIF3 may be required for CycE translation. To explore this possibility, we treated cultured cells with dsRNA targeting eIF3 complex subunits and determined the effect that this had on CycE expression levels. As expected, treating cells with dsRNA targeting CycE transcripts results in a significant reduction in CycE protein levels (Figure 6A). However, treatment of cells with dsRNA targeting eIF3 subunits had no significant effect on CycE protein levels (Figure 6A). This result suggests that the increase in cells with G1 DNA content following RNAi targeting eIF3 subunits is not the result of reduced expression of CycE.

Activation of Cdk2 during G1 results in phosphorylation of proteins that mediate entry of cells into S phase. We examined the possibility that RNAi targeting eIF3 subunits could affect Cdk2 expression or activation of Cdk2/CycE kinase activity. We immunoprecipitated CycE complexes from cells that had been treated with dsRNAs targeting eIF3 subunits and examined their ability to phosphorylate histone H1, a model Cdk2 substrate. We observed a significant decrease in CycE-associated kinase activity for complexes purified from cells treated with dsRNA targeting eIF3 subunits in comparison to cells treated with control dsRNA (Figure 6B). Surprisingly, simultaneously targeting *Dap *rescued CycE-associated kinase activity in these cells even though it did not rescue their ability to efficiently progress from G1 into S phase (Figure 6C and Figure 5). Thus, reduction in CycE-associated kinase activity is not sufficient for the G1 phenotype observed following knockdown of eIF3 subunits. These results suggest that eIF3 is required for a G1 to S rate-limiting process that is independent of Cdk2/CycE activation.

## Discussion

Large-scale RNAi-based screens have begun to play a critical role in defining sets of genes that regulate specific cellular processes [27]. Numerous screens have been completed and new screens are being published at a rapid pace. As the data from these screens accumulates, it becomes increasingly important to understand how to interpret the results that they report particularly in light of the fact that similar screens have shown relatively low levels of overlap. In the current study we re-screened, using a different RNAi library, the genes that were identified as hits in two previous screens for cell cycle regulators, and we were able to confirm many of the original phenotypes. However, our screen also failed to confirm a significant number of genes from both of the original screens. These genes must be considered as potential false positives that may have been originally identified in the previous screens as a result of off-target effects. We failed to confirm 62.6% of the phenotypes for genes screened in common with the genome-wide study (Figure 4A). The rate of off-target effects that we observed in our own screen (39.5%), as defined by phenotypes generated by the first dsRNA that were not confirmed with a second unrelated dsRNA (Figure 4b), suggests that false positives could account for a significant proportion of the unconfirmed phenotypes. Thus, while other factors could contribute to the lack of overlap between screens (e.g. differences in RNAi protocol, knockdown efficiency, cell type), our results suggest that off-targets are an important factor. Our findings again highlight the importance of confirming hits from an initial screen by using multiple dsRNAs. Genes with phenotypes confirmed in our study can now be considered as likely true positive cell cycle regulators. These genes begin to define a complete list of genes that regulate the cell cycle in cultured Drosophila cells and they could be given priority over genes identified only in a single study when determining what genes are to be examined further for their specific roles in regulating cell cycle progression.

In addition to rescreening genes that were hits from previous screens, we also re-screened a subset of the genes that were negatives in the previous screens. We identified the subset of negatives to rescreen by searching the DroID database for protein interaction partners of putative cell cycle regulators. By screening a group of these interactors as well as a group of non-interactors we showed that the rate at which cell cycle phenotypes were identified was significantly higher for interactors than for non-interactors. Confirmation of phenotypes by testing additional dsRNAs indicates that many of these genes were indeed true positives. This means that they were false negatives in the previous screens. These results demonstrate that protein interaction data can be used to guide an RNAi-based screen to be more efficient than a random screen. A similar network-guided approach has been used to predict RNAi knock down phenotypes in *C. elegans *[107,108]. Our results indicate that use of a confidence scoring system to select a higher confidence protein network can improve the performance of this approach (Additional File 6). Our data also support the use of protein interaction data to set thresholds for distinguishing hits from non-hits in large-scale RNAi-based screens, as has been previously suggested [109,110]. Interaction network-guided screening approaches will be particularly important for screens where an undirected, genome-wide approach is not feasible, such as genome-wide combinatorial RNAi screens where pairs of genes are targeted simultaneously.

How are we to interpret the results of large-scale RNAi-based screens in cultured cells? Our data, as well as data from other studies, show that these screens can contain relatively high numbers of false positives and false negatives. Any gene identified as a hit in an initial screen should be considered a potential false positive until it has been confirmed by additional dsRNAs or functional assays. Likewise, genes that are negative in an initial screen remain potential false negatives and the probability that a particular negative is actually a false negative can in part be determined by examining protein interaction data. In light of the presence of both false positive and false negative results in RNAi-based screens, it is not surprising that there is a lack of perfect overlap between the results reported by similar screens. Our results show that retesting genes that were positive in a particular screen can help validate their role in the biological process being examined. In addition, retesting genes that were negative in previous screens can lead to a more comprehensive list of regulators for a particular process.

Our results can be combined with the results of previous studies to identify a set of genes that are confirmed, high-confidence cell cycle regulators in cultured Drosophila cells (Additional File 4). Among the G1 regulators from this list are multiple members of three related protein complexes that are known as the zomes. Although knocking down members of zomes complexes causes a G1 arrest, we showed that the mechanisms responsible for this arrest differ between zomes. The G1 arrest and reduced CycE-associated kinase activity that is induced by targeting subunits of the COP9 signalosome or the proteasome lid is suppressed by simultaneously targeting the Drosophila Cdk2 inhibitor Dap. This suggests that the COP9 signalosome and proteasome lid function under normal conditions to destabilize Dap as has been shown for p27^KIP1 ^in vertebrate cells [97,111,112]. In cultured Drosophila cells, RNAi targeting the signalosome subunit CSN1 results in increased levels of Dap protein [95] providing further evidence that the G1 phenotype observed following signalosome subunit knock down is the result of increased Dap stability. Like the other zomes, targeting eIF3 subunits resulted in G1 arrest. Knock down of eIF3 also reduced levels of CycE-associated kinase activity, which could be rescued by targeting Dap. This is consistent with a possible role for eIF3 in regulating Dap levels, similar to the way that eIF3 regulates p27^KIP1 ^in human cells. Reducing expression of human eIF3a causes an increase in protein levels of p27^KIP1^, while overexpressing eIF3a leads to a decrease in p27^KIP1 ^protein levels [113,114]. Surprisingly, while knock down of Dap rescued CycE-associated kinase activity to normal levels in cells with eIF3 targeted, it did not overcome the G1 arrest. The persistence of the G1 phenotype when eIF3 subunits and Dap are targeted simultaneously indicates that eIF3 knockdown affects G1/S progression through a Cdk2-independent mechanism. It is possible that in addition to Dap, eIF3 regulates genes that function downstream of active Cdk2 in promoting G1/S progression; e.g., eiF3 may regulate genes important for executing initiation of DNA synthesis. Further support for this hypothesis comes from RNAi experiments targeting the human DNA damage response gene TopBP1 [115]. RNAi targeting TopBP1 results in a G1 arrest and activation of the Cdk2 inhibitors p21^CIP1 ^and p27^KIP1^. Simultaneously targeting the Cdk2 inhibitors rescued Cdk2 activity in these cells but did not affect the G1 phenotype, similar to what we observed for eIF3. Interestingly, RNAi targeting TopBP1 led to defects in the loading of replication factors onto DNA demonstrating a mechanism whereby knockdown of genes that function downstream of Cdk2 can result in a G1 arrest phenotype that persists in the presence of active Cdk2.

## Conclusions

Our results indicate that large-scale RNAi-based screens contain significant numbers of both false positives and false negatives. Protein interaction network data can be used to guide re-screening efforts to generate more comprehensive and accurate lists of the genes involved in specific biological processes. In the case of the cell cycle, protein interaction data helped us efficiently identify false negatives from previous screens, while re-screening with secondary dsRNAs validated many genes as authentic cell cycle regulators. Combining our data with data from previous screens allowed us to define a set of high confidence Drosophila cell cycle regulators. Among the high confidence regulators of the G1/S transition we showed that eiF3 complexes regulate the cell cycle by CycE/Cdk2-dependent and independent mechanisms.

## Methods

### Virtual protein-protein interaction screen

The bait proteins used in the virtual protein-protein interaction screen were taken from the published hits that were identified in two large-scale, RNAi-based screens for cell cycle regulators in cultured Drosophila cells [60,61]. Only genes that gave a G1 or G2/M phenotype were taken from the Bjorklund et al. [60] study while all hits were taken from the Bettencourt-Dias et al. [61] study. In addition, the baits included all genes with a Gene Ontology that contained the biological process term "cell cycle". These genes were obtained as a batch download from the Flybase database [116] on 09/26/07. The complete list of 642 baits was used to search DroID, the Drosophila Interactions Database [63] using the dynamic graphing tool, IM Browser [117]. Seven datasets of protein-protein interactions (PPI) from DroID were searched, including data from three large-scale yeast two-hybrid studies [44,118,119], and published data curated by other PPI databases. The remaining three datasets contained interactions predicted for Drosophila proteins based on experimental interaction data for *S. cerevisiae*, *C. elegans*, and human. The relatively high rate of false positives reported for large-scale protein interaction map data [120,121] suggests that not all of the interaction partners that were identified by querying DroID are true interactors. In some cases false positives result from so-called sticky proteins that have many interaction partners in the interaction map [122]. In an effort to reduce the number of false positives, all bait proteins with >30 interactions had their interaction partners removed. Any removed interactors that were identified in more than one independent study were then added back. Finally, all interaction partners for Cdk1 and Cdk2 were added back because of the central role that these proteins play in cell cycle regulation. The results of this analysis identified the set of proteins that we referred to as interactors. By using somewhat arbitrary criteria for selecting interactions (e.g., interactions with baits that had <30 interactors, and inclusion of all Cdk1 and Cdk2 interactors) we effectively included some low confidence interactions. This enabled us to test whether high or low confidence interactions are more useful in the network-guided RNAi screen (Additional File 6). The set of non-interactors was randomly selected from genes in DroID that were in the overall protein interaction map in DroID, but that did not connect with any of the baits or their direct interactors. It is interesting to note that protein interaction data for organisms other than Drosophila performed as well as, or in some cases better than, protein interaction data that is available for Drosophila proteins (Additional File 6 and Additional File 9). Any screening approach that takes advantage of protein interaction data should therefore consider not just interactions available for a particular organism, but all available interaction data.

### Generating double stranded RNAs

Templates available in the Drosophila RNAi Library Release 1.0 and 2.0 (Open Biosystems) were used to generate dsRNA [3,65]. To determine the gene that was targeted by each template in the library we performed BLAST analysis of the predicted amplicon sequences that were provided by the distributor. In some cases, there was no amplicon information provided. For these positions in the library, we obtained amplicon information from the library developer in order to determine the targeted gene [3,65]. Templates were PCR amplified using the library universal primer (5' TAA TAC GAC TCA CTA TAG GGA GAC CAC GGG CGG GT 3') to generate fresh template. 1.5 μl of fresh template was used in *in vitro *transcription (IVT) reactions to generate dsRNAs targeting baits, interactors, and non-interactors. Control templates targeting the GFP gene were generated by amplification of GFP template DNA using a T7-containing primer pair (5'-GAA TTA ATA CGA CTC ACT ATA GGG AGA TGC CAT CTT CCT TGA AGT CA-3', and 5'-GAA TTA ATA CGA CTC ACT ATA GGG AGA TGA TGT TAA CGG CCA CAA GTT-3'). Templates were arrayed into 96-well PCR plates with 1 to 3 GFP templates/plate and 6 μl IVT reactions were performed using either MegaScript (Ambion) or Ampliscribe (Epicentre Biotechnologies) T7 kits according to the manufacturer's instructions. dsRNA was purified from IVT reactions using MultiScreen_HTS_-96-well filter plates (Millipore) in conjunction with a Biomek NX MC Laboratory Automation Workstation. 95 μl of nuclease-free water was added to each IVT reaction before transfer onto the filter plate. Reactions were passed through the filter plate by application of vacuum pressure for 35 minutes. dsRNA was eluted from the filter into 110 μl of nuclease-free water by shaking at 1100 rpm for 10 minutes. Purified dsRNAs were analyzed by spectrophotometry and stored at -80°C.

### Cell Culture, RNA interference, and Flow Cytometry

Drosophila S2R+ cells were obtained from the Drosophila Genomics Resource Center (Indiana University) and cultured in Schneider's media (Invitrogen) supplemented with 10% Fetal Bovine Serum and 0.1 mg/ml Gentamicin (GIBCO). For induction of RNAi, cells were re-suspended in Schneider's media at a density of 4 × 10^5 ^cells/ml and 75 μl of cell suspension was added per well to duplicate 96-well cell culture plates that had been spotted with 5 μl of dsRNA per well. Cells were incubated for 1.5 hours before addition of 150 μl of Schneider's media containing 10% fetal bovine serum and 0.1 mg/ml gentamicin. After a 6-day incubation period, media was removed from cells and cellular DNA was stained by adding Schneider's media containing 2 μl/ml Vybrant DyeCycle Orange stain (Invitrogen). Cells were dislodged from the growth surface by pipetting and incubated at room temperature in the dark for 1 hour. Cell cycle profiles were obtained by analyzing cells on a FACSArray flow cytometer (BD Biosciences). The percentage of cells with G1, G2/M, greater than G2/M and subG1 DNA content was determined using the BD FACSArray system software with user-defined gates for each measured parameter.

### Data Analysis

In order to normalize the data across plates, a global average (*g*) for each measured parameter was calculated using all non-GFP well values and a plate average (*p*) was calculated for each measured parameter using all non-GFP well values for each plate. Raw data on each plate was normalized by multiplying the raw data by the factor *g*/*p*. Normalized averages for each dsRNA were determined by calculating the average of the normalized duplicate values. A mean of the normalized values from wells treated with GFP dsRNA was calculated. This mean was used to calculate standard deviations from the mean of normalized values for each non-GFP dsRNA. A dsRNA was considered a hit (i.e., affecting cell cycle progression), if the percentage of cells in a phase was >3 standard deviations from the mean for that phase. Gene Ontology analysis of hits was done using the GO term enrichment tool DAVID [67] and data in the GO database release 2010-10-30 [62].

### Validation dsRNAs

For 258 of the genes that scored as hits in the primary screen, we generated templates for preparing a minimally overlapping (0-11nt) validation dsRNA. Amplicon sequences from the Open Biosystems library were compared with amplicon sequences from the Drosophila RNAi Screening Center (DRSC) RNAi library [25]. If amplicons showed overlap of <12 nucleotides, primer pairs for generating the DRSC amplicon were ordered. If no DRSC amplicon with <12 nucleotide overlap was available, primer pairs for generating a minimally overlapping amplicon were manually designed. Templates for generating dsRNA were prepared using the DRSC or manually designed primer pairs and a 2-step PCR approach. The first step PCR reaction was performed with primers containing a GC rich anchor 5'GGGCGGGT3' and the products of this reaction were amplified in the second step PCR reaction using the T7-containing universal primer (above). dsRNA generation, cell treatment, data acquisition and analysis were all performed as described above for the primary screen.

### Combinatorial RNAi

Drosophila S2R+ cells were re-suspended in Schneider's Media at a density of 4 × 10^5 ^cells/ml and 75 μl of cell suspension was added to 96-well cell culture plates that had been spotted in triplicate with 5 μl of dsRNA targeting individual zomes subunits and 5 μl of dsRNA targeting GFP or Dacapo. Cellular DNA was stained and flow cytometry was performed as described above.

### Western Blot

Drosophila S2R+ cells were re-suspended in Schneider's media at a density of 6 × 10^6 ^cells/ml and 1.5 ml of cell suspension was added to 25 cm^2 ^cell culture flasks. 20 μg of dsRNA was added to cells and they were incubated at 25°C for 1.5 hours before addition of 3 ml Schneider's media containing 10% FBS and 0.1 mg/ml gentamicin. Following a 6-day incubation, cells were resuspended in 1× RIPA lysis buffer (Cell Signaling) and placed on ice for 30 minutes. Cells were further disrupted by 10 needle strokes through a 21-gauge needle. Clarified cell lysates were collected, run on SDS-PAGE, then immunoblotted using anti-Cyclin E antibody 1:1000 (Santa Cruz SC-33748), anti-beta-tubulin 1:5000 (E7 antibody) or anti-alpha-tubulin 1:10000 (Sigma B-5-1-2).

### Immunoprecipitation and Kinase Assays

Drosophila S2R+ cells were re-suspended in Schneider's media at a density of 2.3 × 10^6 ^cells/ml and 1.5 ml of cell suspension was added to 25 cm^2 ^cell culture flasks. 20 μg of dsRNA was added to cells and they were incubated at 25°C for 1.5 hours before addition of 3 ml Schneider's media containing 10% FBS and 0.1 mg/ml gentamicin. Following a 6-day incubation, cells were dislodged from the growth surface and resuspended in lysis buffer (0.2% NP-40, 200 mM Tris-HCl, 200 mM NaCl, 100 mM Na_2_EDTA, 20 mM EGTA, 200 mM NaF, Na_3_VO_4_, 100 mM glycerol phosphate, 40 mM PMSF, 1× Protease Inhibitor Cocktail [123]) and incubated on ice for 30 minutes. Clarified lysates were collected and stored at -80°C. For immunoprecipitation, 10 μl of anti-Cyclin E antibody (Santa Cruz sc-33748) was added to 400 μg of protein extract and incubated 30 minutes at room temperature with agitation. 20 μl of Protein A-agarose (Santa Cruz sc-2001) was added to immunoprecipitations and tubes were placed at 4°C with agitation overnight. Immunoprecipitations were pelleted and washed twice with kinase assay buffer (10 mM MgCl_2_, 10 mM DTT, 50 mM Hepes pH7.5). After the second wash, pellets were resuspended in 13 μl kinase assay buffer. To initiate the kinase assay reaction, 0.2 μg of histone H1 in kinase assay buffer and 5 μl of [γ^32^P]-ATP (1.0 μCi/μl) was added. Kinase reactions were incubated 30 minutes at room temperature before being terminated by the addition of gel loading buffer.

## Authors' contributions

STG designed and carried out the experiments. JY carried out bioinformatic and computational analyses. DL examined gene expression in S2R+ cells. JAH and MAK contributed to the RNAi screens. RLF helped design and interpret all experiments and co-wrote the paper with STG. All authors read and approved the final manuscript.

## Supplementary Material

Additional file 1 **Baits and their interaction partners from DroID.** This file contains information for the genes that were used as baits in our virtual protein interaction screen as well as a list of the interactions that were discovered by querying DroID.  Both the unfiltered and filtered sets of interactions are shown. Click here for file

Additional file 2 **Baits are more significantly connected to each other in protein-protein interaction data than are random sets of proteins**. (A) Number of protein-protein interactions between the proteins that were used as baits for the virtual protein-protein interaction screen (red dot) as compared to random groups of proteins containing the same number of nodes. A PPI network was generated for each of 1000 random groups of proteins (see below) by searching DroID for interactions. The number of within-group interactions was counted and plotted for each network. (B) Size of the largest connected subnetwork component for the proteins used as baits (red dot) as compared to random groups of proteins containing the same number of nodes.  As in (A), an interaction network was generated for each of 1000 random groups of proteins (see below). The largest connected subnetwork component for each network is the number of nodes in the largest subnetwork of nodes that are connected either directly or indirectly in each network. For both A and B the random groups of proteins were selected to have the same distribution of interactions/protein (degree) as the baits.  To do this we counted the number of baits with degrees in four ranges and found that 374 baits had degrees from 1-49, 99 baits had degrees from 50-99, 75 baits had degrees from 100-199, and 37 baits had degrees from 200-982. Each random group of proteins was selected by randomly sampling from all proteins in the interaction data the same number of nodes from each of the four degree ranges; e.g., 374 with degrees from 1-49, 99 with degrees from 50-99, and so on. 1000 such random groups were independently sample for each figure A and B. Click here for file

Additional file 3 **Data table for the initial screen, validation screen and repeat screen**. Raw and normalized data and gene information for each amplicon used in the initial screen, validation screen, and repeat screen. Click here for file

Additional file 4 **Initial screen hits, validation screen hits, and high confidence regulators**. Gene information for the initial screen hits, the validated hits, and the high confidence regulators. Click here for file

Additional file 5 **Genes that displayed a phenotype are more highly connected to the baits than are genes that did not display a phenotype**. The number of interactor-bait interactions for each protein classified as an interactor in the unfiltered interaction data.  The graph shows a comparison between the interactors that displayed a phenotype in the initial screen (hits) versus those that did not (non-hits). The box plots show median values (horizontal line) and 25th and 75th percentiles in the box below and above the line, respectively, while the whiskers show the 91st and 9th percentile. Click here for file

Additional file 6 **Performance of protein interaction data sets in guiding discovery of novel cell cycle regulators**. The chart shows the percentage of interactions between a bait and an interactor where the interactor was a validated hit from the screen.  The percentage is based on all bait-interactor interactions from each indicated data set (dark grey) or only high confidence bait-interactor interactions (light grey). The high confidence interactions are those with confidence scores >0.5 as determined in [66].  All datasets are from DroID.  The “Drosophila” dataset includes only experimentally measured protein-protein interactions (PPI).  Other datasets (S. cerevisiae, C. elegans, and human) are predicted PPI based on experimental detection of interactions with orthologous proteins from the indicated organisms. Click here for file

Additional file 7 **Cell cycle gene enrichment in the RNAi screens**. The chart shows the percentage of the genes from each data set that are annotated with a Gene Ontology [62] biological process of cell cycle (GO: 0007049). The level of enrichment for the cell cycle term, relative to all Drosophila genes, was determined by the GO term enrichment tool, DAVID [67] located at http://david.abcc.ncifcrf.gov/home.jsp.  p-values are enrichment scores calculated by DAVID with a modified Fisher’s exact test. q-values are p-values corrected for multiple testing using the false discovery rate (FDR) method [67]. Click here for file

Additional file 8 **Suppression of G1 arrest by simultaneous knockdown of Dacapo is protein complex-specific**. S2R+ cells were treated with dsRNAs targeting the indicated members of the COP9 signalosome or eIF3 protein complex (A), or the proteasome lid complex (B) in combination with either dsRNA targeting GFP (light grey) or Dacapo (dark grey).  The percentage of cells with G1 DNA content was determined by flow cytometry. Click here for file

Additional file 9 **High confidence protein interaction data contains a higher percentage of bait-bait interactions**. The chart shows the percentage of bait-bait interactions from each of the indicated protein interaction data sets (dark grey) or only the high confidence data from each indicated data set (light grey). The high confidence interactions are those with confidence scores >0.5 as determined in [66]. Click here for file
