# Supplementary figures and images for: A protein network-guided screen for cell cycle regulators in Drosophila
Source: BMC Syst Biol. 2011 May 6;5:65. doi: 10.1186/1752-0509-5-65 (PMC3113730; doi:10.1186/1752-0509-5-65)

## Additional File 2.

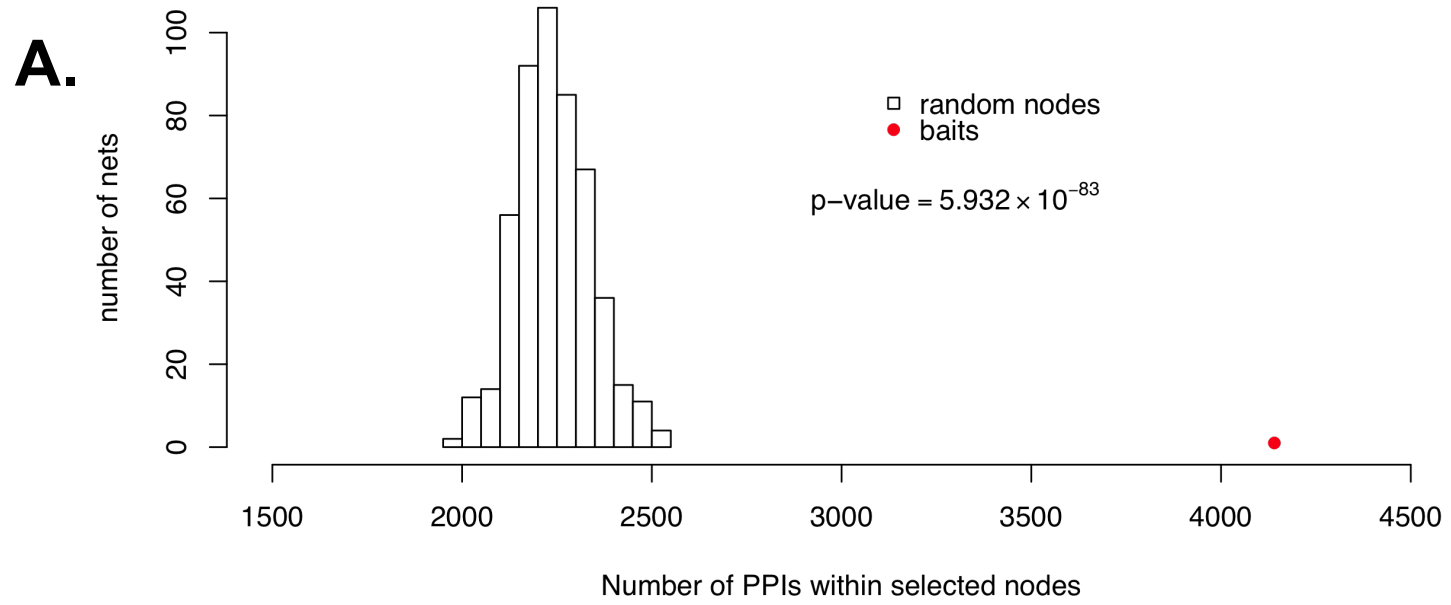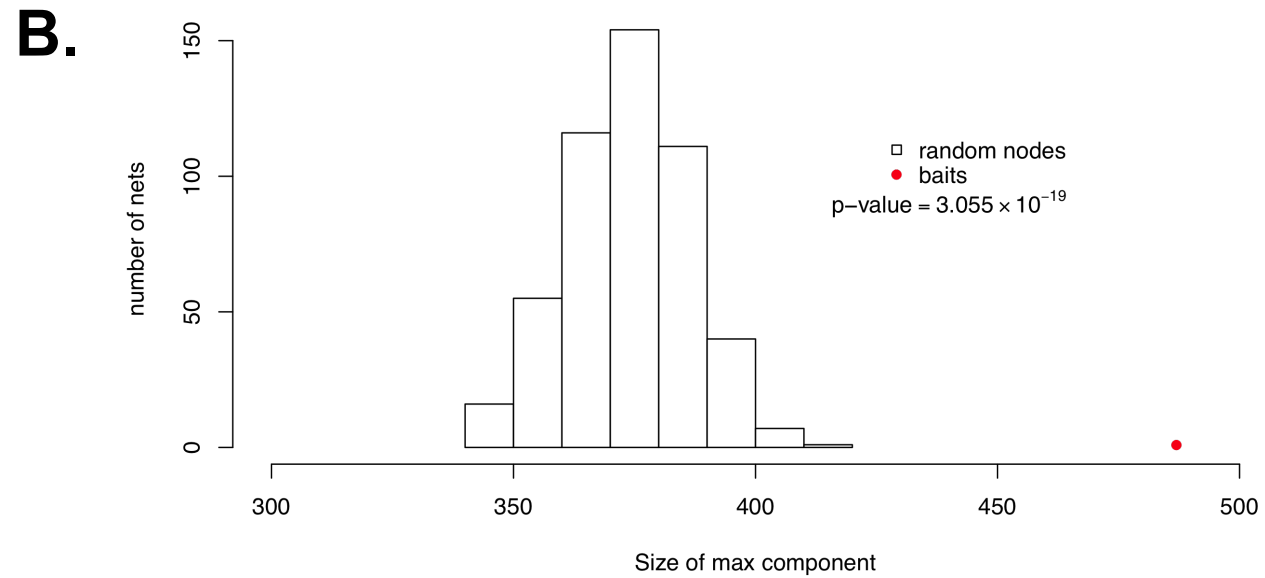

Supplement: Additional file 2 — Baits are more significantly connected to each other in protein-protein interaction data than are random sets of proteins. (A) Number of protein-protein interactions between the proteins that were used as baits for the virtual protein-protein interaction screen (red dot) as compared to random groups of proteins containing the same number of nodes. A PPI network was generated for each of 1000 random groups of proteins (see below) by searching DroID for interactions. The number of within-group interactions was counted and plotted for each network. (B) Size of the largest connected subnetwork component for the proteins used as baits (red dot) as compared to random groups of proteins containing the same number of nodes. As in (A), an interaction network was generated for each of 1000 random groups of proteins (see below). The largest connected subnetwork component for each network is the number of nodes in the largest subnetwork of nodes that are connected either directly or indirectly in each network. For both A and B the random groups of proteins were selected to have the same distribution of interactions/protein (degree) as the baits. To do this we counted the number of baits with degrees in four ranges and found that 374 baits had degrees from 1-49, 99 baits had degrees from 50-99, 75 baits had degrees from 100-199, and 37 baits had degrees from 200-982. Each random group of proteins was selected by randomly sampling from all proteins in the interaction data the same number of nodes from each of the four degree ranges; e.g., 374 with degrees from 1-49, 99 with degrees from 50-99, and so on. 1000 such random groups were independently sample for each figure A and B. [file 1752-0509-5-65-S2.PDF]

## Additional File 5.

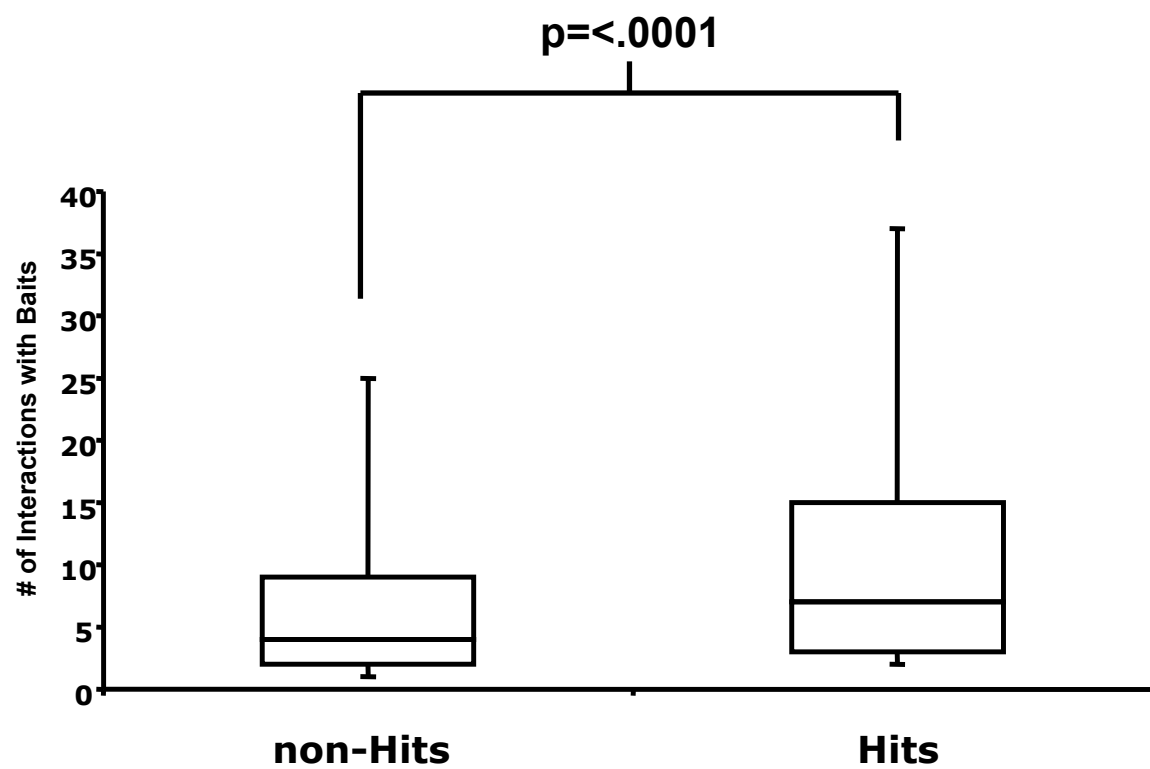

Supplement: Additional file 5 — Genes that displayed a phenotype are more highly connected to the baits than are genes that did not display a phenotype. The number of interactor-bait interactions for each protein classified as an interactor in the unfiltered interaction data. The graph shows a comparison between the interactors that displayed a phenotype in the initial screen (hits) versus those that did not (non-hits). The box plots show median values (horizontal line) and 25th and 75th percentiles in the box below and above the line, respectively, while the whiskers show the 91st and 9th percentile. [file 1752-0509-5-65-S5.PDF]

## Additional File 6.

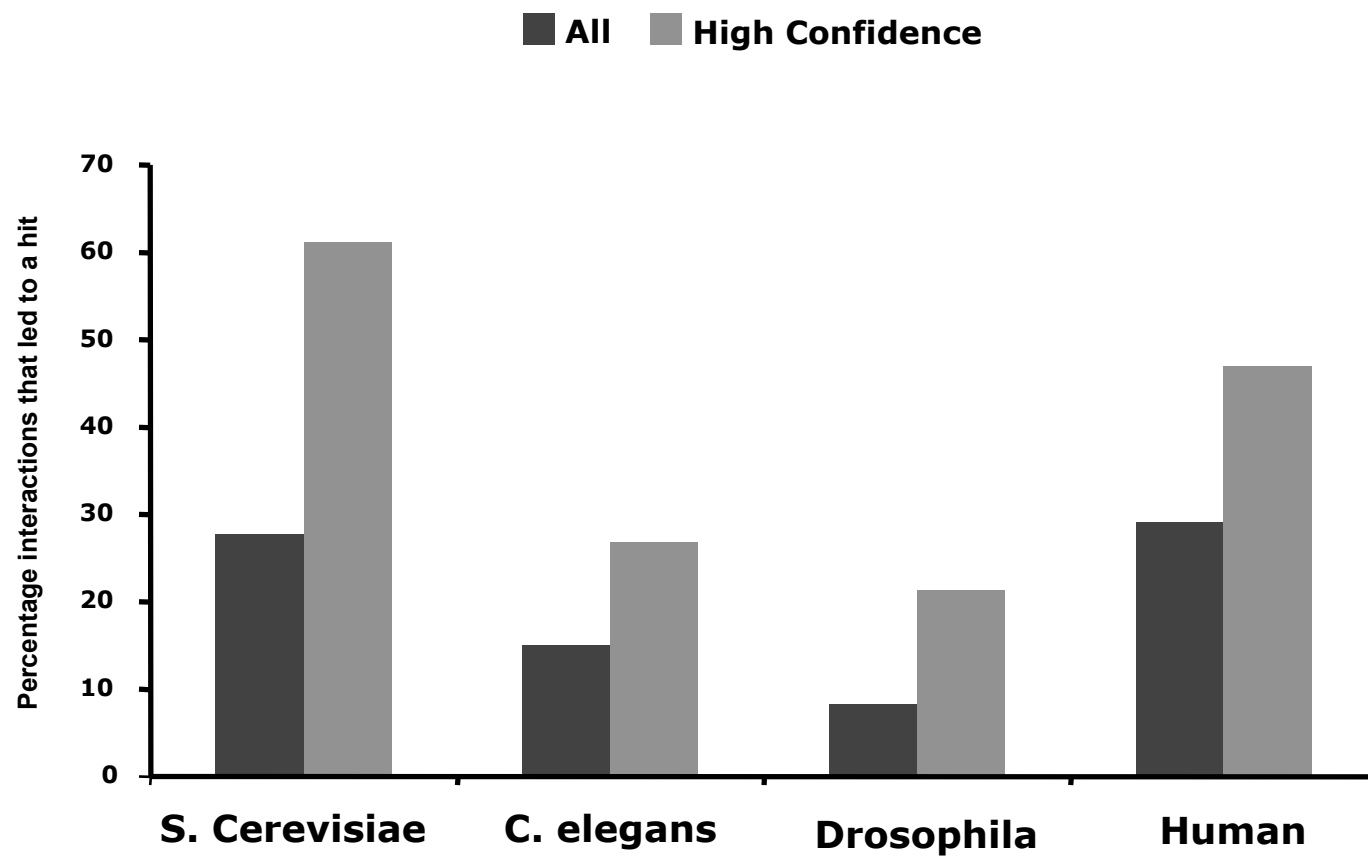

Supplement: Additional file 6 — Performance of protein interaction data sets in guiding discovery of novel cell cycle regulators. The chart shows the percentage of interactions between a bait and an interactor where the interactor was a validated hit from the screen. The percentage is based on all bait-interactor interactions from each indicated data set (dark grey) or only high confidence bait-interactor interactions (light grey). The high confidence interactions are those with confidence scores >0.5 as determined in [66]. All datasets are from DroID. The “Drosophila” dataset includes only experimentally measured protein-protein interactions (PPI). Other datasets (S. cerevisiae, C. elegans, and human) are predicted PPI based on experimental detection of interactions with orthologous proteins from the indicated organisms. [file 1752-0509-5-65-S6.PDF]

## Additional File 7.

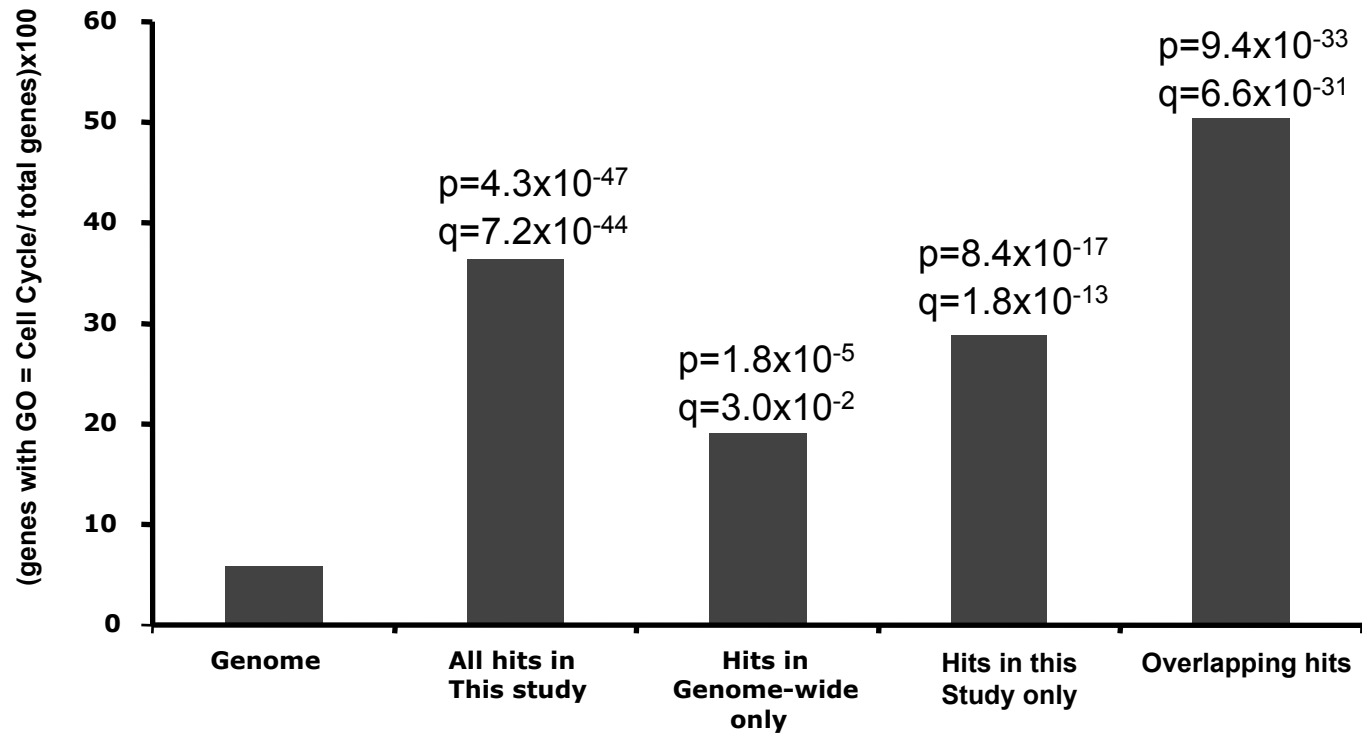

Supplement: Additional file 7 — Cell cycle gene enrichment in the RNAi screens. The chart shows the percentage of the genes from each data set that are annotated with a Gene Ontology [62] biological process of cell cycle (GO: 0007049). The level of enrichment for the cell cycle term, relative to all Drosophila genes, was determined by the GO term enrichment tool, DAVID [67] located at http://david.abcc.ncifcrf.gov/home.jsp. p-values are enrichment scores calculated by DAVID with a modified Fisher’s exact test. q-values are p-values corrected for multiple testing using the false discovery rate (FDR) method [67]. [file 1752-0509-5-65-S7.PDF]

## Additional File 8.

**A.**

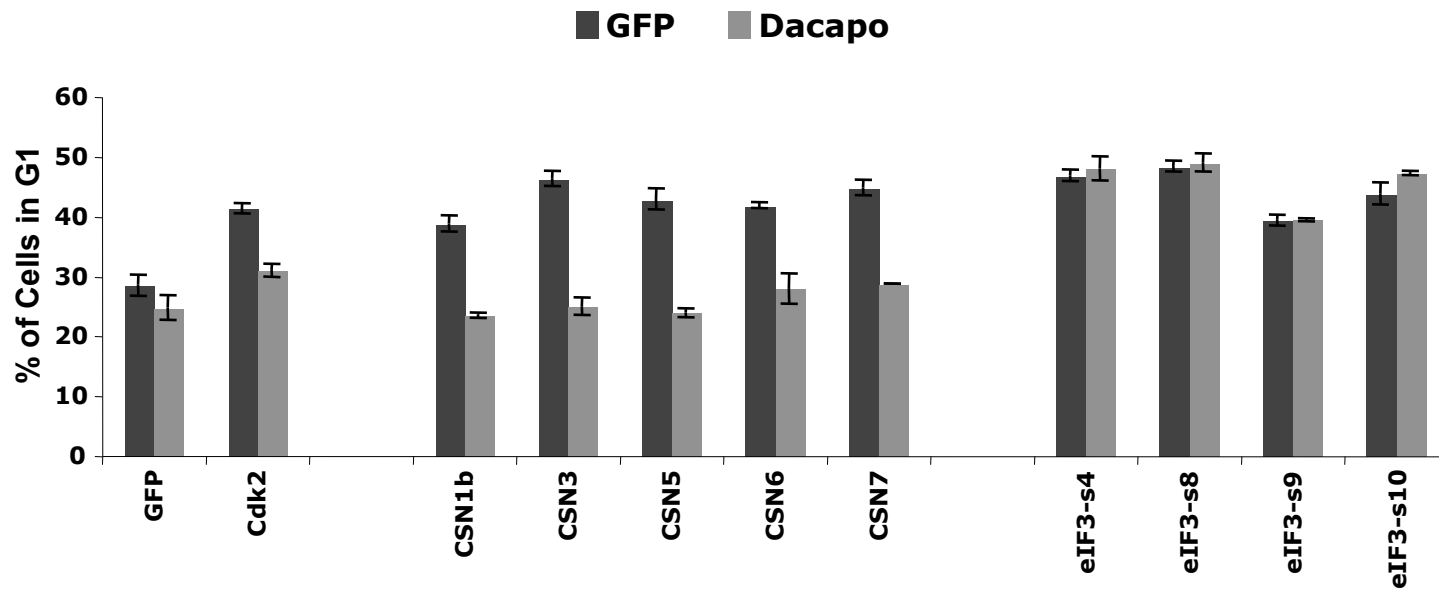

**B.**

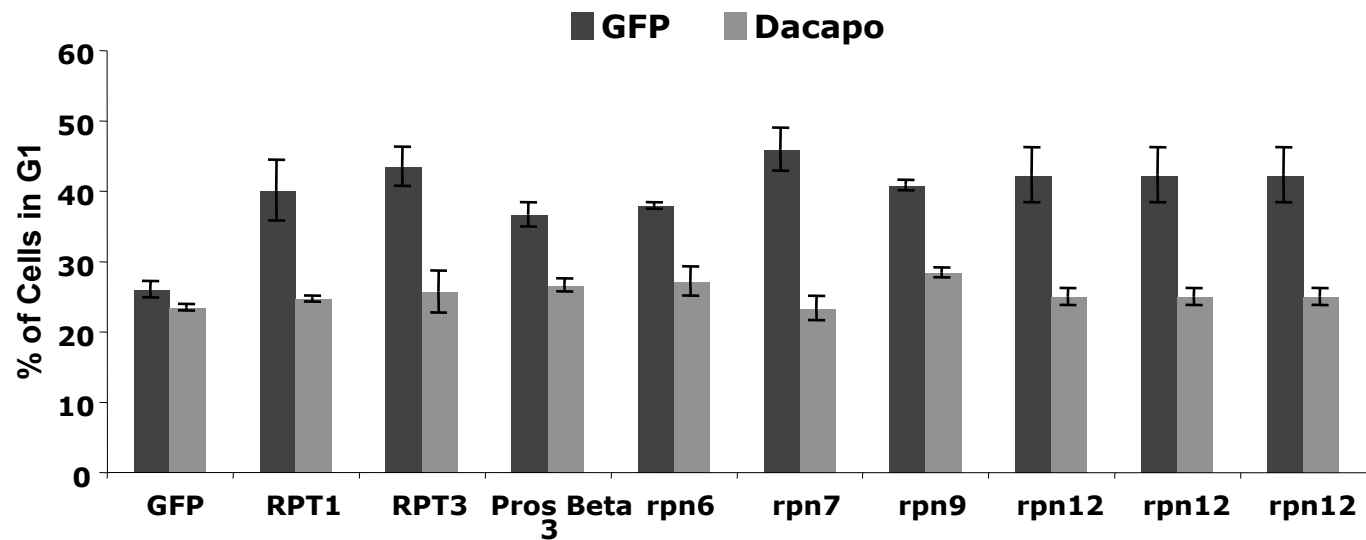

Supplement: Additional file 8 — Suppression of G1 arrest by simultaneous knockdown of Dacapo is protein complex-specific. S2R+ cells were treated with dsRNAs targeting the indicated members of the COP9 signalosome or eIF3 protein complex (A), or the proteasome lid complex (B) in combination with either dsRNA targeting GFP (light grey) or Dacapo (dark grey). The percentage of cells with G1 DNA content was determined by flow cytometry. [file 1752-0509-5-65-S8.PDF]

## Additional File 9.

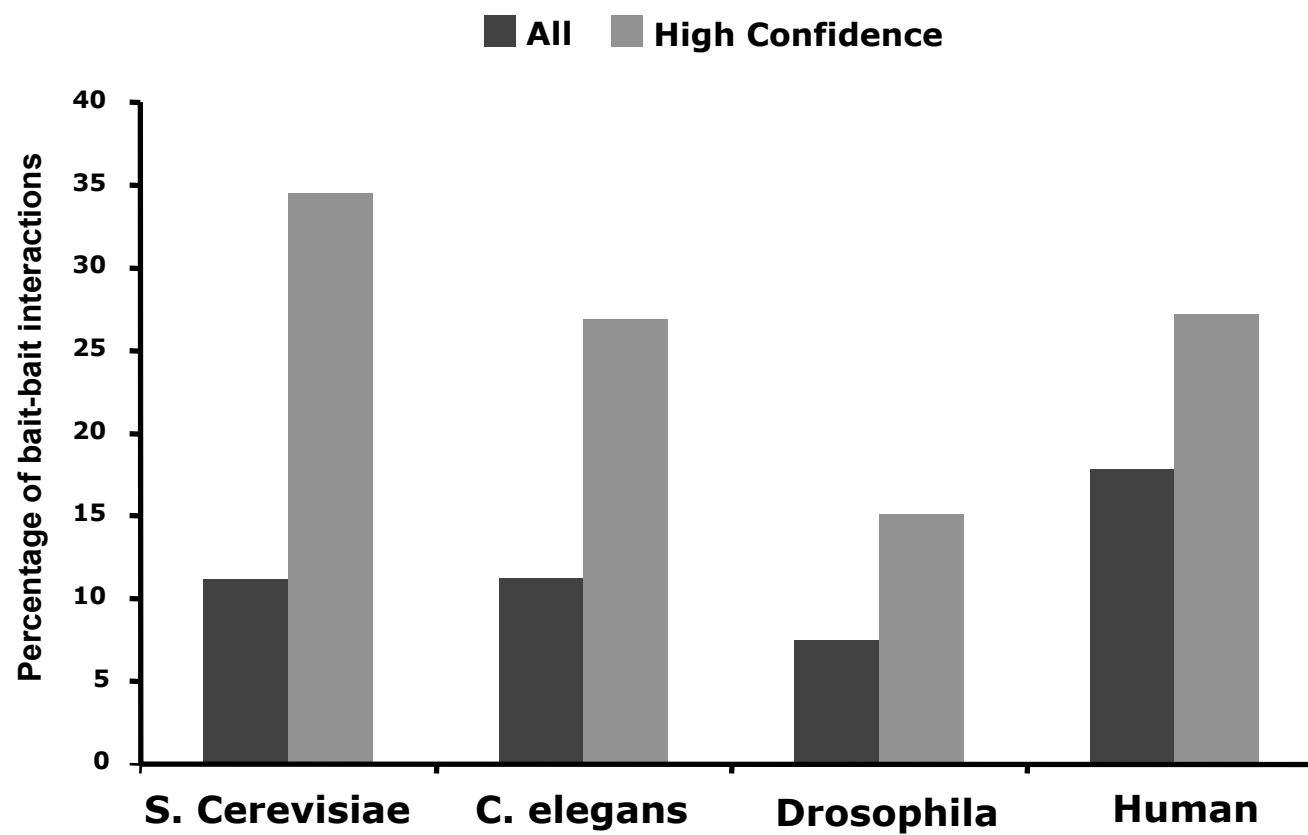

Supplement: Additional file 9 — High confidence protein interaction data contains a higher percentage of bait-bait interactions. The chart shows the percentage of bait-bait interactions from each of the indicated protein interaction data sets (dark grey) or only the high confidence data from each indicated data set (light grey). The high confidence interactions are those with confidence scores >0.5 as determined in [66]. [file 1752-0509-5-65-S9.PDF]
